# Biomechanical investigation of dynamic hip screw and wire fixation on an unstable intertrochanteric fracture

**DOI:** 10.1186/s12938-019-0663-0

**Published:** 2019-04-24

**Authors:** Hsu-Fu Wu, Chih-Han Chang, Gwo-Jaw Wang, Kuo-An Lai, Chung-Hwan Chen

**Affiliations:** 1 Department of BioMedical Engineering, National Cheng Kung University, Tainan, Taiwan; 2 Orthopaedic Research Center, College of Medicine, Kaohsiung Medical University, Kaohsiung, Taiwan; 3 Department of Orthopaedics, College of Medicine, Kaohsiung Medical University, Kaohsiung, Taiwan; 4 Department of Orthopaedic Surgery, University of Virginia, School of Medicine, Charlottesville, VA 22908 USA; 5 National Ciao Tung University, Hsinchu, Taiwan; 6 Department of Orthopedics, National Cheng Kung University Hospital, College of Medicine, National Cheng Kung University, Tainan, 701 Taiwan; 7 Orthopaedic Research Center, Kaohsiung Medical University, No 100 Shih Chuan 1st road, SanMing district, Kaohsiung, Taiwan; 8 Department of Orthopaedics, College of Medicine, Kaohsiung Medical University, No 100 Shih Chuan 1st road, SanMing district, Kaohsiung, Taiwan; 9 Division of Adult Reconstruction Surgery, Department of Orthopedics, Kaohsiung Medical University Hospital, Kaohsiung Medical University, No 100 Tzu-You 1st road, SanMing district, Kaohsiung, Taiwan; 10 Department of Orthopedics, Kaohsiung Municipal Ta-Tung Hospital, Kaohsiung Medical University, Kaohsiung, No 68 ZhongHua 3rd road, Cianjin district, Kaohsiung, Taiwan

**Keywords:** Dynamic hip screw, Intertrochanteric hip fracture, Lesser trochanteric fragment, Wire, Unstable type

## Abstract

**Background:**

Although use of a dynamic hip screw (DHS) for stable intertrochanteric hip fracture fixation has been successfully applied in fracture healing for more than 20 years, DHS fixation on unstable intertrochanteric fractures still has a high failure rate, especially in patients with osteoporosis. Although the wire fixation is usually incorporated with orthopedic device to treat fracture, the wiring techniques are developed through experiences. Thus, this study is objective to investigate the biomechanical property of different wire fixation methods incorporated with DHS system to provide the lesser trochanter fragment stable fixation on osteoporotic TypeA2.1 fracture for enhancing stability after bone reduction.

**Results:**

Sawbone testing results demonstrated higher maximum load, stiffness, and energy in a DHS with wire fixation compared with DHS fixation only. In static biomechanical testing of a cadaver femur, we compared the stiffness of five fixation models and then tested a fatigue failure model in cycle loading with DHS fixation only. Wiring fixation can enhance stability and the cut-out failure model in the fatigue test was identical to the clinical failure model.

**Conclusions:**

Lesser trochanteric fragment fixation is a crucial concern in the stability of an A2.1 unstable fracture, and the combination of a wiring technique with a DHS seems beneficial for achieving better stability. The addition of an antirotational greater trochanter is likely to enhance stability through wiring of the greater trochanter.

## Introduction

Osteoporotic fractures are a major cause of morbidity in the older population. Taiwan has the highest hip fracture rate in Asia, with incidence rates of 392 per 100,000 in women and 196 per 100,000 in men [1]. Hip fractures are composed of femoral neck and pertrochanteric fractures. Intertrochanteric fractures account for approximately 45% to 50% of all hip fractures in the elderly and 50–60% are classified as unstable. The total annual cost of hip fractures also is expected to double in that time, to $16 billion [2]. The principal treatment for hip fracture in older patients is surgery [3]. Most femoral neck fractures are treated using arthroplasty, whereas regarding pertrochanteric fractures, injuries such as intertrochanteric fractures require internal fixation [4]. The in-hospital and 1-year mortality rates are as high as 9.5% and 14–36%, respectively [5–7]. Patients with hip fractures also exhibit a high incidence of comorbidities, which majorly affect mortality. The presence of three or more comorbidities is the strongest preoperative risk factor for mortality in patients with hip fractures [8]. Therefore, a high quality of surgical fixation for intertrochanteric fractures facilitates patient care with early wheelchair transfer, and walking with a walker may affect the outcomes of hip fracture patients by decreasing the risk of pressure and pneumonia.

Intertrochanteric fractures are classified as stable and unstable fractures according to the fracture fragment and direction of the fracture line [9]. A stable intertrochanteric fracture is a two-part fracture with a fracture line along the trochanter line, whereas an unstable intertrochanteric fracture is one where comminution of the posteromedial buttress exceeds a trochanteric fragment or where the fracture lines are within the subtrochanter [10]. Clinical results have indicated that the conventional DHS can provide beneficial stability for simple and nonosteoporosis fractures but is unable to provide stable fixation for unstable or osteoporotic intertrochanteric fractures. Although use of DHS for stable intertrochanteric hip fracture fixation has been successful in fracture healing for more than 20 years, DHS fixation on unstable fractures has a failure rate of 3–26% [11–15], especially in osteoporotic fractures. Because the posteromedial buttress is the most crucial supporting point in load bearing [16], a single DHS fixation cannot provide stable fixation of a lesser trochanter fragment in an unstable intertrochanteric fracture. Supplemental fixation of the posteromedial buttress is required in unstable intertrochanteric fractures.

Cerclage wiring is a simple technique that has been employed extensively since the advent of surgical treatment for fractures. Many studies have reported a wide range of results and clinical applications for various cerclage techniques. With additional wire fixations, unstable intertrochanteric fractures can be sufficiently stabilized using DHS until bone union is achieved [17–19]. Although the wire fixation is usually incorporated with orthopedic device to treat fracture, the wiring techniques are developed through experiences. Thus, the purpose of this research is to investigate the biomechanical property of different wire fixation methods incorporated with DHS system to provide the lesser trochanter fragment stable fixation on osteoporotic TypeA2.1 fracture for enhancing stability after bone reduction.

## Materials and methods

### Sawbone static biomechanical testing

**Fig. 1:**
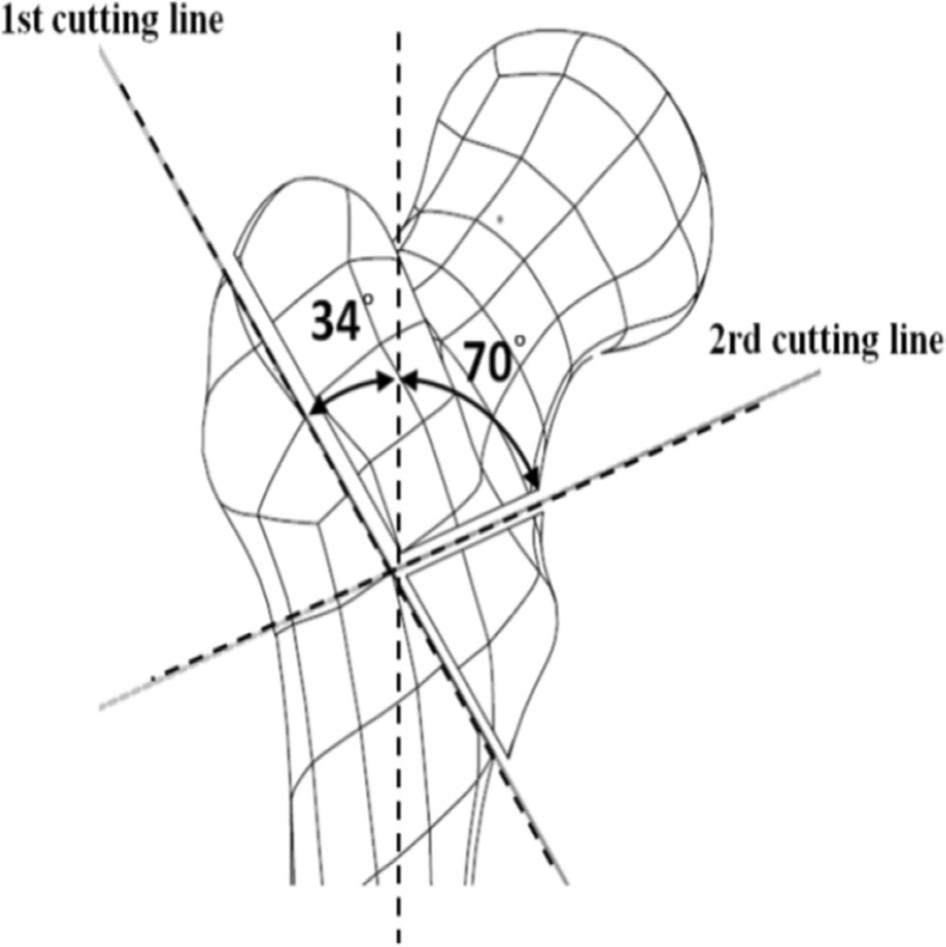
A2.1 fracture model of the femur sawbone

**Fig. 2:**
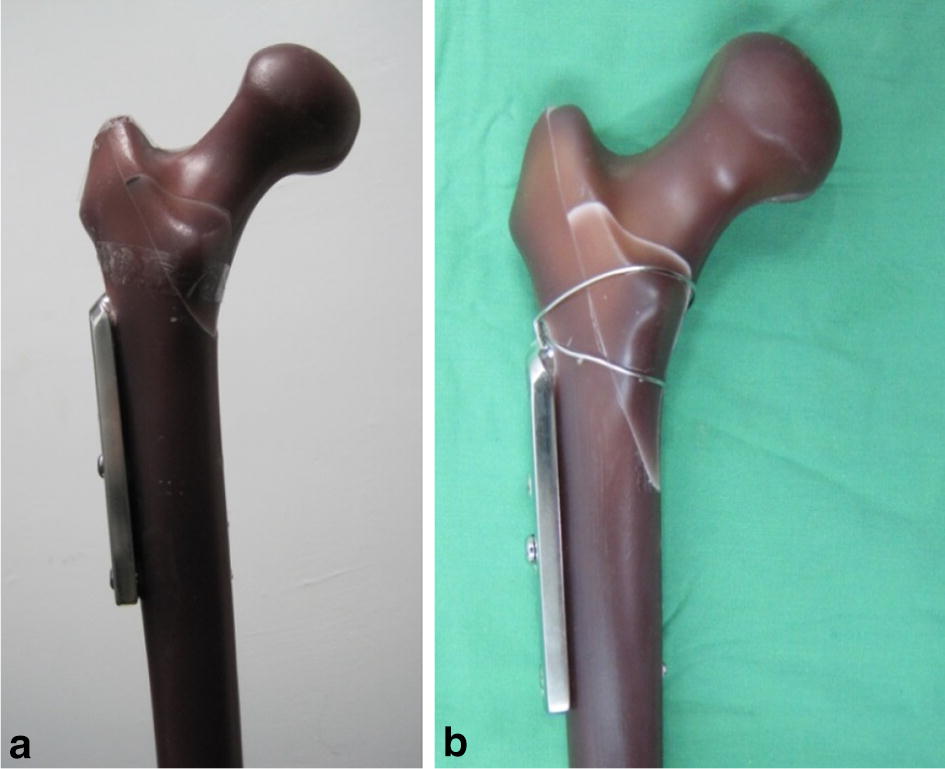
**a** DHS fixation only, **b** wire incorporated DHS fixation

Six sawbones (#3103, Sawbones Inc., USA), six set of DHS fixation systems (Synthes, four holes), and cerclage wire (Synthes, 1.4 mm, stainless steel) were used in this study. A femur sawbone model with an unstable intertrochanteric fracture (A2.1) was established in accordance with the American Orthopaedic Trauma Association classification [20]. All sawbones were clamped in a sawing template and the osteotomy was performed using a line saw machine. The intertrochanteric osteotomy was divided into two steps. The first step was to saw along the line at an anticlockwise 34° angle to the femoral shaft, and the second step was to saw along the line at a clockwise 70° angle to the femoral shaft, as depicted in Fig. 1. In the wiring fixation method, two wire coils were used—a lower wire coil passing the lesser trochanter inferior to the greater trochanter inferior to fix the lesser trochanter fragment, and an upper wire coil passing the lesser trochanter superior to the greater trochanter inferior to balance the muscle traction on the lesser trochanter superior (Fig. 2). The lag screw was positioned centrally along the femoral head in both the lateral and anteroposterior (AP) views, and the tip of the screw was 10 mm from the articular surface [21].

**Fig. 3:**
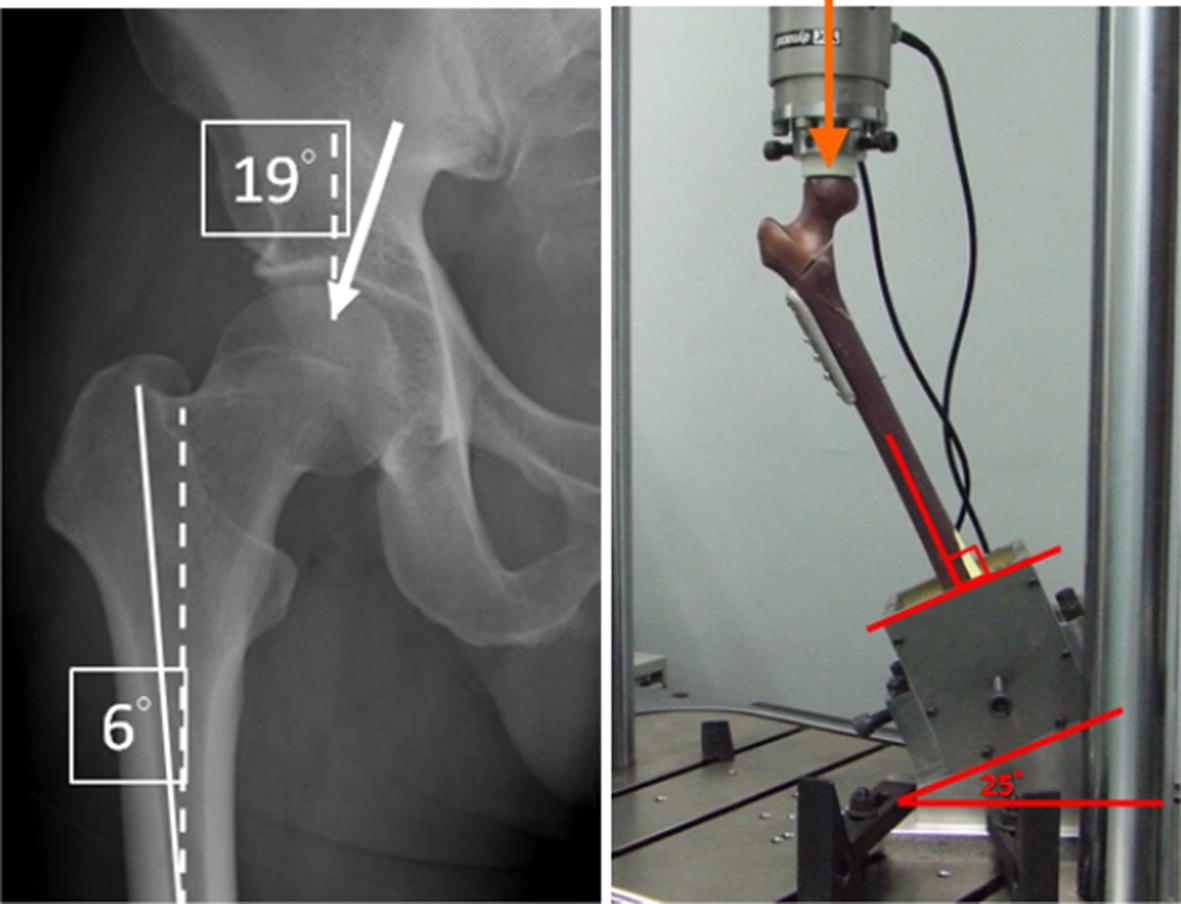
Illustration of femur mounting position and force direction

An experiment was conducted on the A2.1 fracture with DHS fixation to investigate the stability of the intact structure with and without wiring. A universal material testing machine (Instron, ElectroPuls E10000, UK) was used to apply a load to identify failures where the femur had broken or a “cut-out” had occurred. To simulate the direction of hip joint force during a single-leg stance, the femurs were mounted in a gypsum fixture with 25° adduction on the table of the material testing machine [22–24], as presented in Fig. 3. Mechanical testing was performed using the universal testing machine and a load was applied to the femoral head through a ultrahigh molecular weight polyethylene (UHMWPE) cup.

Loading was performed in two stages. The first stage was to apply a 100-N preload at a strain rate of 2.5 mm/min, and the second stage involved increasing the strain rate to 10 mm/min. Testing was completed after a bone fracture occurred on the femur or the displacement reached 25 mm. Load and displacement until failure were recorded on a 15-kHz computer-based data acquisition system. Stiffness and maximum load force were determined through recording force and displacement to evaluate their stability.

### Cadaver static and dynamic biomechanical testing

**Fig. 4:**
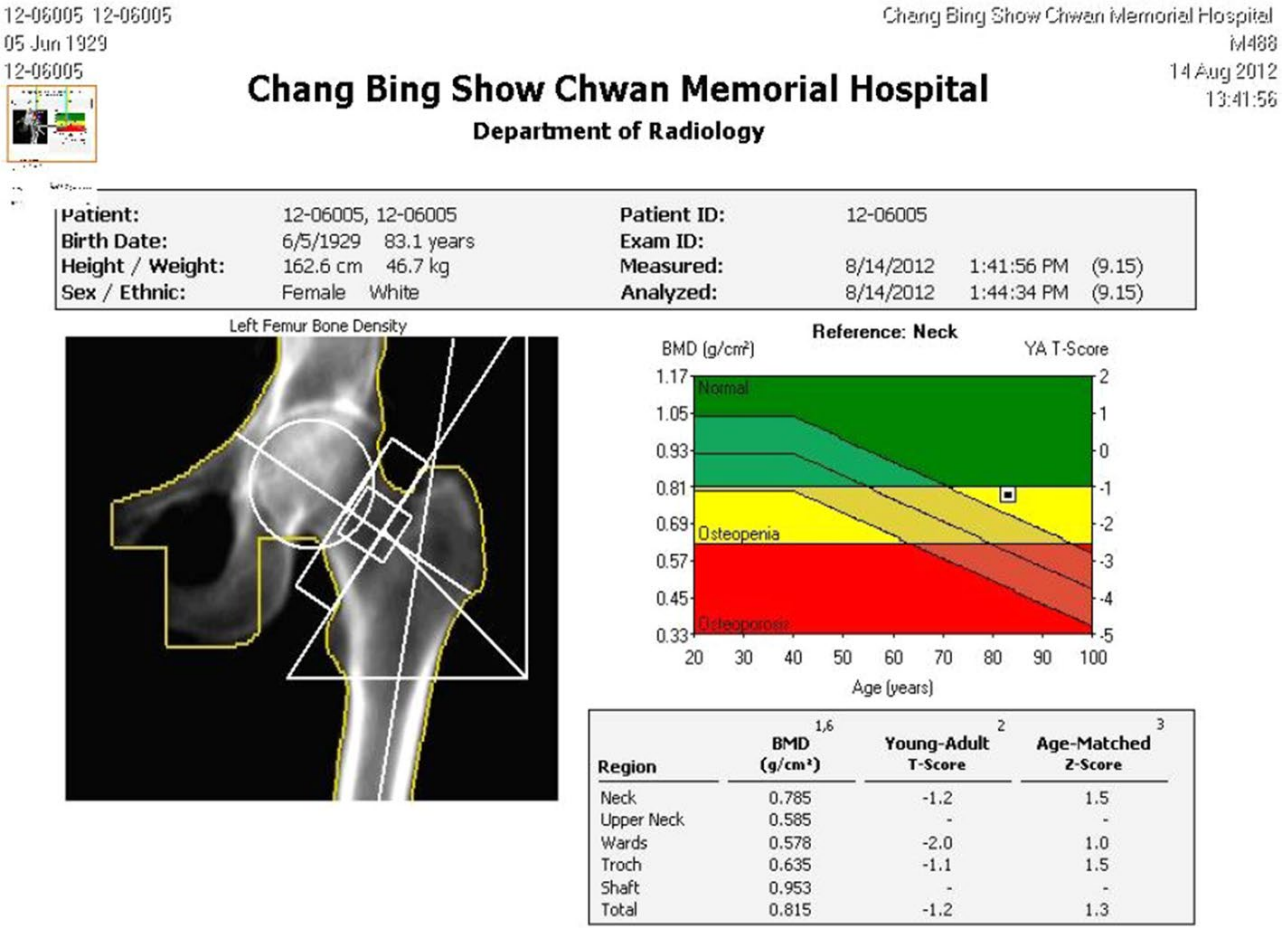
DXA-based information regarding the cadaver

**Fig. 5:**
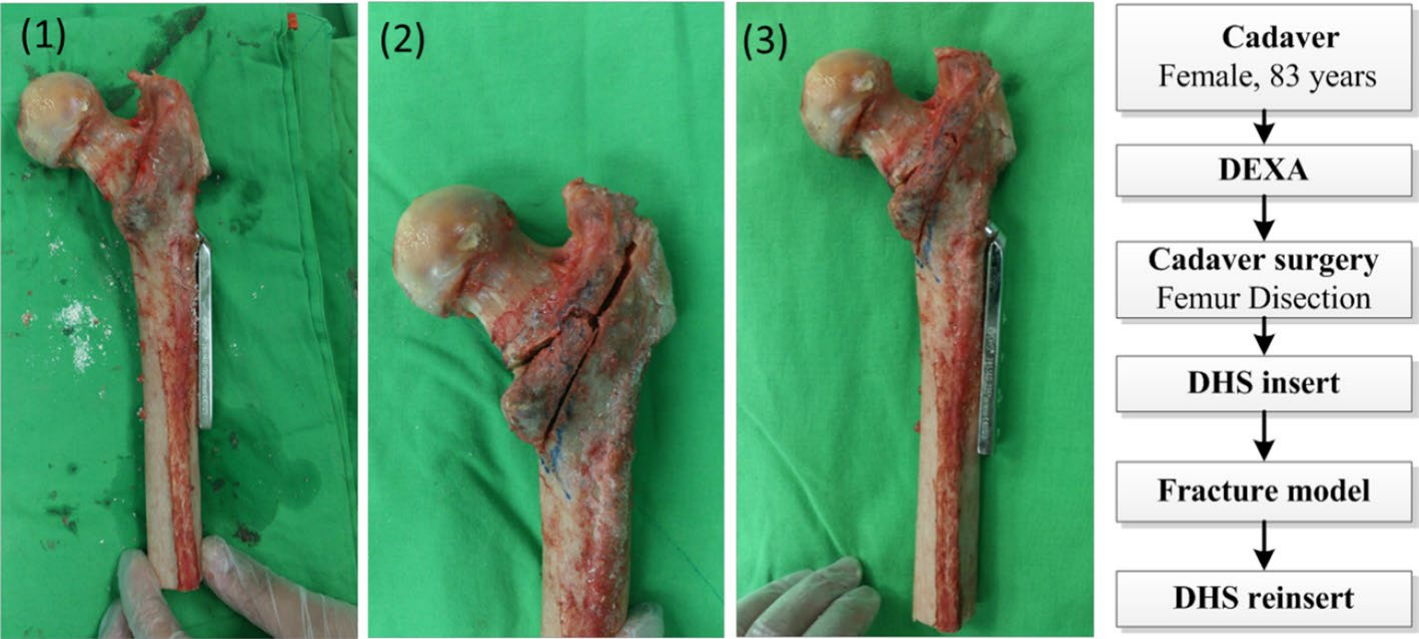
Cadaver specimen preparation procedure: (**1**) insert and then remove the DHS; (**2**) create a fracture model; (**3**) reinsert the DHS

A human femur from an 83-year-old female was used in this study. Bone mineral density (BMD) was measured using dual-energy X-ray absorptiometry (DXA) (Fig. 4), and based on the BMD of the upper neck, wards, troch, and shaft, the femur was defined as osteopenia (Before testing, the specimens were maintained at 4 °C overnight, and dissection commenced after 3 h of storage at room temperature (20 °C). The procedure of the DHS inset is described as follows (Fig. 5). (1) Insert the DHS into the cadaver’s femur and remove the DHS. (2) Use an orthopedic saw to create an A2.1 fracture model. (3) Reinsert the DHS into the cadaver’s femur.

**Fig. 6:**
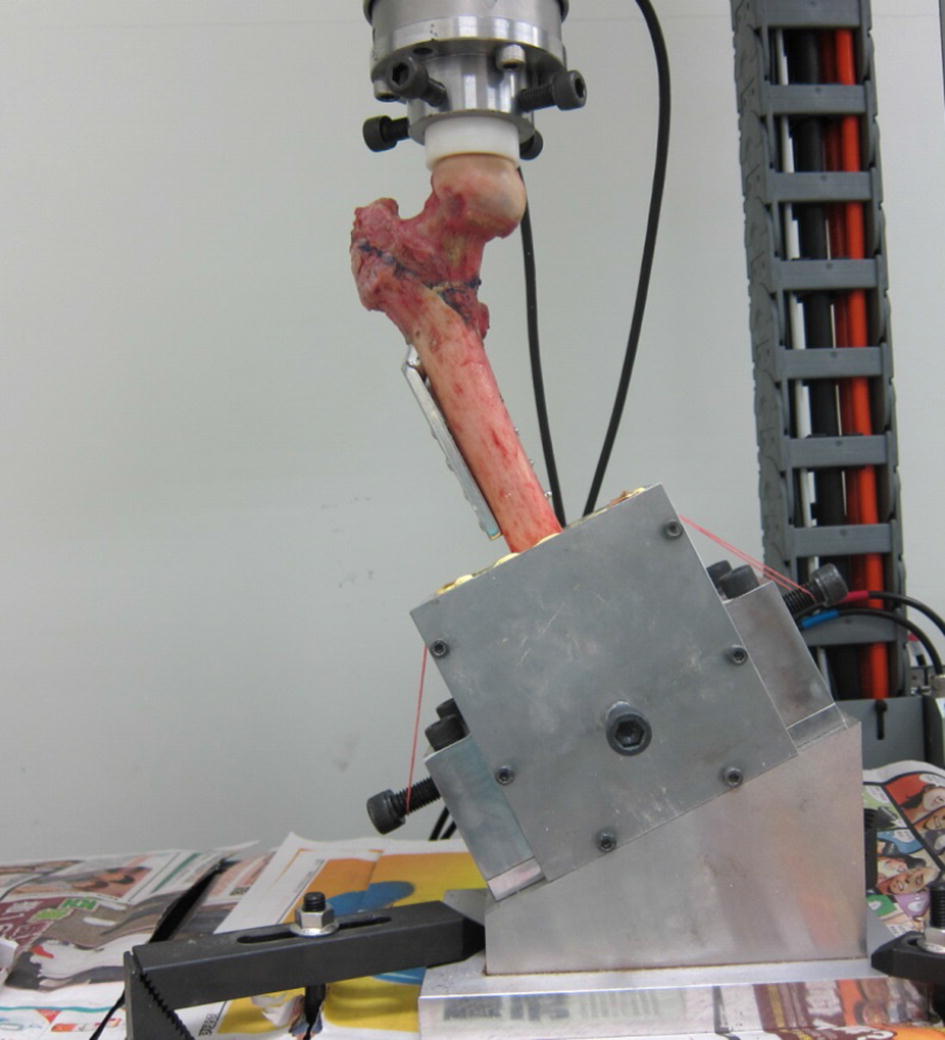
Setup of femur in universal material testing machine

The lag screw was positioned centrally along the femoral head in both the lateral and AP views, and the tip of the screw was 10 mm from the articular surface, as observed using X-ray imaging [21]. To simulate the direction of the hip joint force during a single-leg stance, the femur was mounted in a gypsum fixture with 25° adduction on the table of the materials testing machine [22–24], as presented in Fig. 6.

The test involved two stages. The first stage was to investigate the stability of the five wiring methods in a static test, and the second stage was to analyze fatigue failure in the dynamic test. In static testing, we compared the following five models: (1) DHS fixation only, (2) DHS fixation with one wire coil passing the lesser trochanter inferior to the greater trochanter inferior to provide fixation in the lesser trochanter fragment and ease of operation; (3) DHS fixation with two wire coils—a lower wire coil passing the lesser trochanter inferior to the greater trochanter inferior to fix the lesser trochanter fragment, and an upper wire coil passing the lesser trochanter superior to the greater trochanter inferior to balance muscle traction on the lesser trochanter superior; (4) DHS fixation with three wire coils—a lower wire coil passing the lesser trochanter inferior to the greater trochanter inferior to fix the lesser trochanter fragment, an upper wire coil passing the lesser trochanter superior to the greater trochanter inferior, and one wire coil passing the great trochanter superior to the assisted plate fixed by the first cortical screw to balance muscle traction on the lesser trochanter superior and provide the head fragment part with antirotational resistance force; and (5) DHS fixation with one wire coil passing the great trochanter superior to the assisted plate fixed by the first cortical screw to provide the head fragment part with antirotational resistance force and ease of operation. The testing machine used was the same as that used in the sawbone biomechanical test. Initially, we applied a 50-N preload at a strain rate of 2.5 mm/min, and in the second stage, we increased the strain rate to 10 mm/min. Testing was halted when the load reached 200 N. In dynamic testing, a sinusoid waveform cyclical compressive load of 120–1200 N with a frequency of 1 Hz was applied to the specimen.

## Results

### Sawbone biomechanical testing

**Fig. 7:**
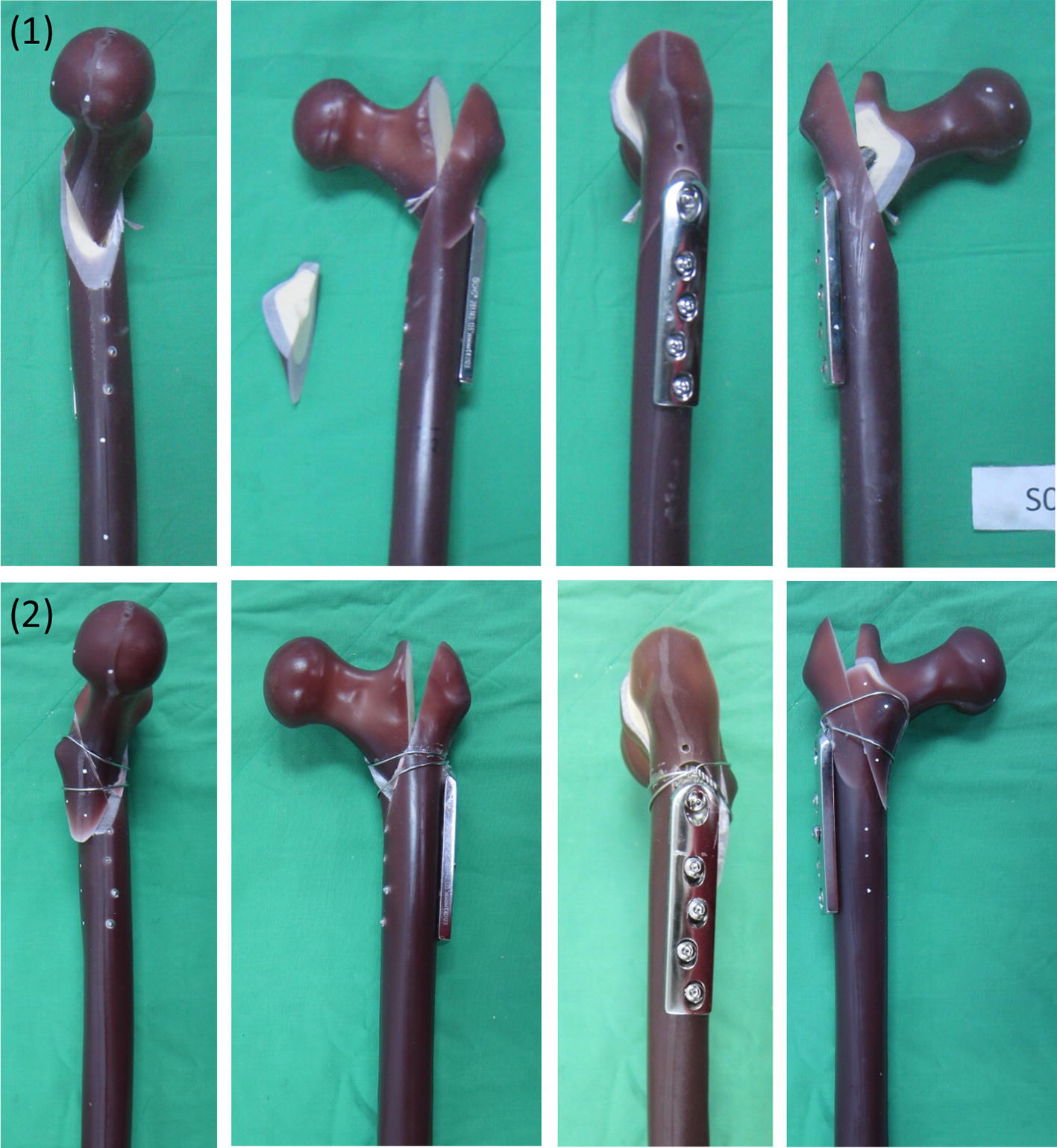
Loading failure specimen: (**1**) DHS fixation only; (**2**) DHS with wire fixation

**Fig. 8:**
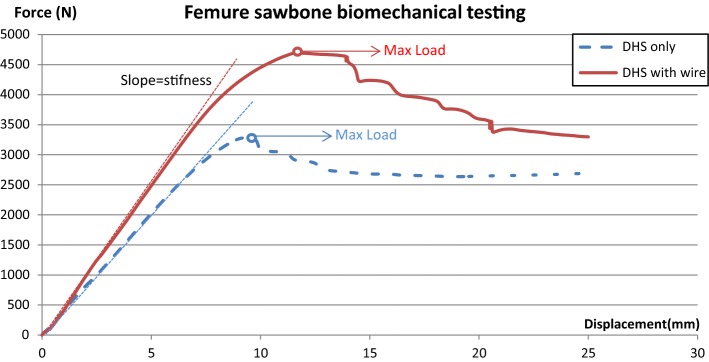
Example of displacement–force curve

The failure results are provided in Fig. 7. A crack appeared in the insert hole of the side plate and the head fragment had a rotational movement in the DHS fixation only and DHS with wire experiments. Examples of the typical load–displacement curves for the DHS fixation only and DHS with wire fixation specimens are presented in Fig. 8. The stiffness is the slope of the linear line fitting the data with *R*^2^ = 0.999. In addition, we calculated the yielding point in the offset linear line of 0.1 mm. According to the analysis results of the force–displacement curve (Table 1), the wiring technique—assisted DHS yielded a significantly higher load for the DHS with the wire model (4514 ± 266 N) compared with DHS fixation only (3310 ± 77 N, *p* = 0.0007). This corresponds to a mean increase in maximum load of 36% for DHS and wire fixation. The difference in displacement of maximal load was not significant between DHS fixation only (9.6 ± 0.6 mm) and DHS with wire (11.0 ± 0.8 mm, *p* = 0.067). The wiring technique assisted DHS resulted in a significantly higher stiffness for DHS with wire model (560 ± 87.3 N) compared to DHS fixation only (395 ± 7.5 N, *p* = 0.01). Though there was no significant difference between yielding force (*p* = 0.839) and the displacement (*p* = 0.273), while the energy calculated the area from original point to the Max. load was higher in the DHS with wire specimen (29.6 ± 3 J) than that in the DHS fixation only specimen (17.6 ± 1.8 J, *p* = 0.003).

**Table 1:** Femur sawbone static testing results

	Max. load (N)	Displacement in Max. load (mm)	Stiffness (N/mm)	Yield force (N)	Displacement in yield force (mm)	Energy (J)
No1	3261.5	8.81	393.7	2960	7.5	15.4
No2	3265.3	9.62	384.8	2965	6.8	17.9
No3	3424.5	10.27	402.1	2924	7.7	19.7
No4	3290.6	9.51	398.5	2895	7.5	17.4
DHS only (= 4)	3310 ± 77	9.6 ± 0.6	395 ± 7.5	2936 ± 33	7.4 ± 0.4	17.6 ± 1.8
No1	4702.1	11.6	498.4	3883	7.90	31.70
No2	4326.1	10.4	621.9	1699	2.84	27.4
DHS with wire (*n* = 2)	4514 ± 266*	11.0 ± 0.8	560 ± 87.3*	2791 ± 1544	5.4 ± 3.6	29.6 ± 3.0*
*p* value	0.001	0.067	0.012	0.839	0.273	0.003

* indicate significant values (*p* < 0.05)

*t* test

### Static and dynamic biomechanical testing on cadaver

**Fig. 9:**
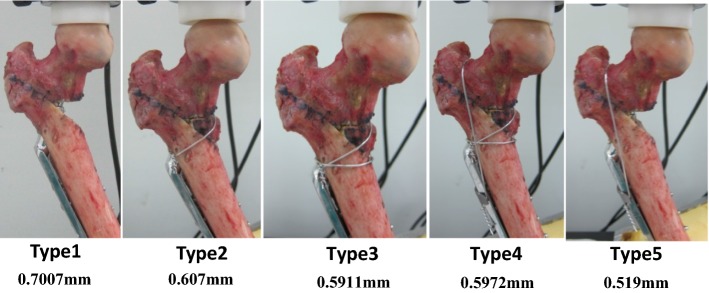
Displacement of cadaver in 200-N loading

**Fig. 10:**
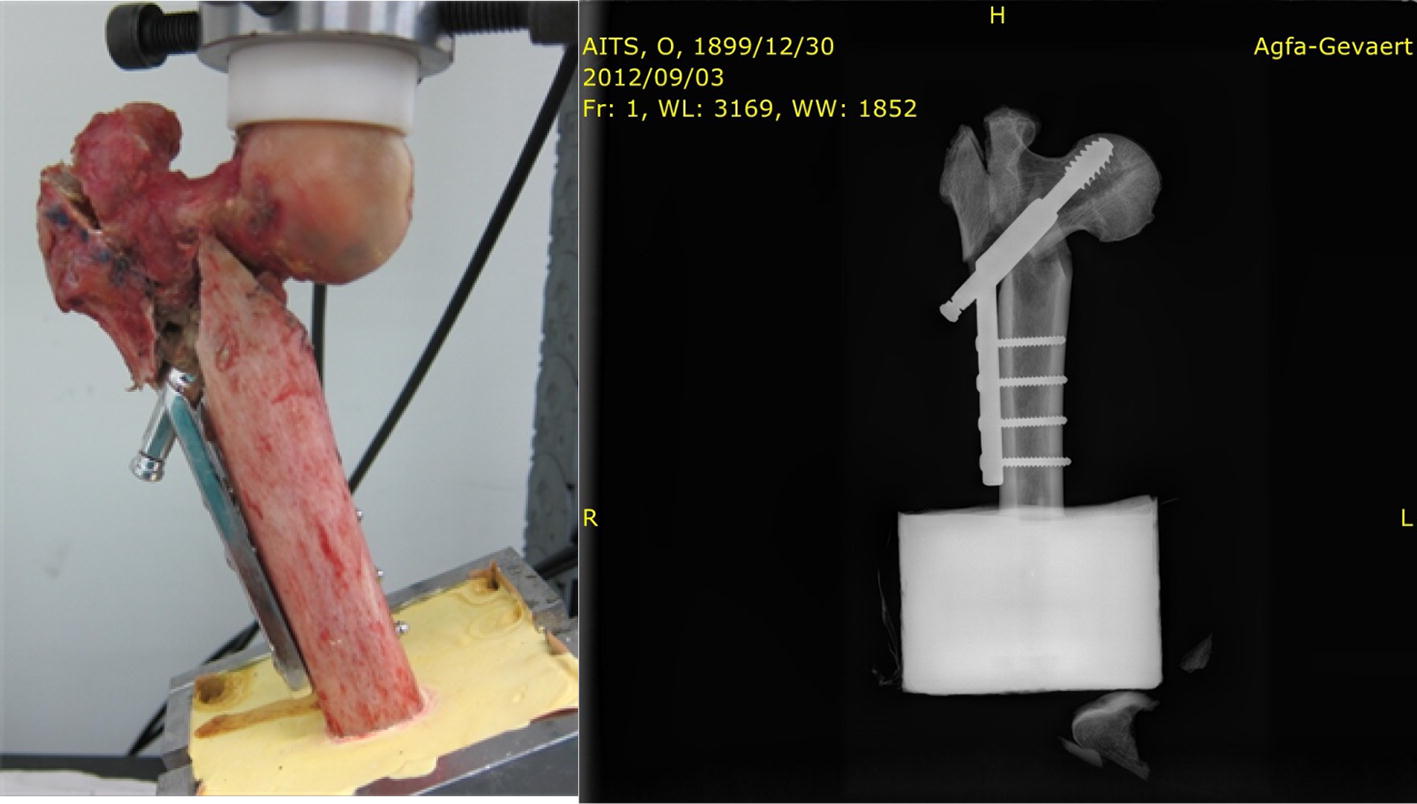
Fatigue failure (cut-out) of femur after cycle loading

In static testing, the displacement results of the DHS with wire fixation were lower than those of DHS fixation only, as presented in Fig. 9. The wiring technique enhanced the stability of DHS fixation in the unstable intertrochanteric fracture. The cadaver’s femur exhibited failure in a 557-iteration cycle loading in dynamic testing. The fracture appeared in the great trochanter inferior and along the lag screw. Additionally, the femoral head fragment was rotated in the observed position. X-ray imaging revealed that the lag screw had no deformation but exhibited relative movement in the femoral head in the common failure model (cut-out) of unstable intertrochanteric fracture in clinical practice (Fig. 10).

## Discussion

The higher Max force means the structure can bear more force and the higher energy means the structure can also bear more energy. Stiffness result was the extent to which femur head displacement in response to an applied force, the more displacement means the more unstable fixation. In sawbone testing, wire fixations were purposed to restore the posteromedial buttress by restricting less trochanter fragment with wire. After restoration of posteromedial buttress by wire fixation, significant increase in stiffness can explain that the integrity of less trochanter fragment is an important buttress. This indicates that only DHS fixation is unlikely to provide stable fixation for a type A2.1 unstable proximal femoral fracture because of poor posteromedial buttress. Restoration of a lesser trochanter fragment can improve posteromedial buttress to provide better stability. Yielding force representative the beginning of material destruction, and the most weak of this structure should be sawbone, so there was no significant difference in yielding force with the same sawbone property. Stiffness is representative of the structure stability before the yielding force, and stiffness increase can explain the enhancement of stability after wiring fixation. The yielding force in the DHS with wire fixation model exhibited a considerable variation due to the relaxation of the wire coil; however, Max. load, stiffness, and energy remained higher than that in the DHS fixation only group. In unstable intertrochanteric fracture, wire coil provides the tighten strength for fracture reduction. When the loading force is greater than the tighten strength provided by wire, relaxation of the wire coil occurs. Relaxation of the wire coil means failure of the fixation because the sliding shear has more force than the fixation of wire. The yielding force was calculated form load–displacement curve, and the wire relaxation is also a kind of material destruction.

We infer that the fixation of the lesser trochanter fragment is a crucial factor in unstable intertrochanteric fractures. Based on our sawbone study, we initially anticipated that two wire coil passing the less trochanter inferior to the great trochanter would result in better outcome. Although more wires in the fixation can provide greater stability, they increase the difficulty of surgery. In cadaver testing, four developed wire fixation techniques combined with DHS created the potential to biomechanically enhance the stability of an unstable intertrochanteric fracture through the wire fixed on the lesser trochanter fragment or greater trochanter fragment. Moreover, the greater trochanter fixation with a wire can resist the rotational movement by applying force, and is beneficial for stability. During dynamic testing of unstable osteoporotic intertrochanteric fractures, a varus momentum occurs. Lack of posteromedial buttress will lead to stress concentration in implants and then femur head cutting-out happened. Restoration of posteromedial buttress can dissipate and reduce stress around implant which can reduce the risk of implant failure. In the other failure model of dynamic testing on the femur, the fracture in the greater trochanter was caused by the applied force, which generated varus movement.

This study had several limitations. First, we did not evaluate the effect of the iliopsoas muscle on the lesser trochanter. The failure model may vary with the effects of the iliopsoas muscle. Second, the strength of the sawbone is not equal to the strength of a bone with osteoporosis. The failure model may be different in the sawbone if these strengths were not equal. Third, we did not use a tensor in the wire application; therefore, we are not certain that the wires were having equal tension in all experiments. This may have led to some variation in the failure model.

## Conclusion

The novel contribution was a comparison between the biomechanical properties of existing wire fixation methods developed through experiences to provide a simple and cheaper wire-assisted DHS fixation technique to enhance the stability in unstable intertrochanteric femur fracture. The combination of a wiring technique and a DHS seems beneficial for improving the stability of an A2.1 unstable intertrochanteric fracture with loss of the posteromedial buttress. This technique enables biomechanically stable construction involving wiring of the lesser trochanter fragment or the greater trochanter. Since there is not much difference between type 2 and type 3, we recommend one wire coil passing above the lesser trochanter and inferior to the greater trochanter can provide fixation in the lesser trochanter fragment and ease of operation. The fracture model can be used to investigate the biomechanical property of different implant fixation systems in unstable intertrochanteric fracture in the future.

## Abbreviations

DHS: dynamic hip screw
AP: anteroposterior
UHMWPE: ultrahigh molecular weight polyethylene
BMD: bone mineral density
DXA: dual-energy X-ray absorptiometry
